# Effects of Glyphosate-, Glufosinate- and Flazasulfuron-Based Herbicides on Soil Microorganisms in a Vineyard

**DOI:** 10.1007/s00128-018-2438-x

**Published:** 2018-09-18

**Authors:** Karin Mandl, Clemens Cantelmo, Edith Gruber, Florian Faber, Barbara Friedrich, Johann G. Zaller

**Affiliations:** 1 Federal College and Research Center for Viticulture and Pomology, Wienerstraße 74, 3400 Klosterneuburg, Austria; 2 Institute of Zoology, University of Natural Resources and Life Sciences, Vienna, Gregor-Mendel-Straße 33, 1180 Vienna, Austria

**Keywords:** Soil biota, Soil microbes, Non-target effects, Pesticide, Viticulture, Weed control

## Abstract

In a vineyard we examined the effects of broad-spectrum herbicides with three different active ingredients (glyphosate, glufosinate, flazasulfuron) on soil microorganisms. Mechanical weeding served as control treatment. Treatments were applied within grapevine rows and soil samples taken from there in 10–20 cm depth 77 days after application. Fungi were analyzed using classical sequencing technology and bacteria using next-generation sequencing. The number of colony-forming units (CFU) comprising bacteria, yeasts and molds was higher under flazasulfuron compared to all other treatments which had similar CFU levels. Abundance of the fungus *Mucor* was higher under flazasulfuron than glufosinate and mechanical weeding; *Mucor* was absent under glyphosate. Several other fungi taxa were exclusively found under a specific treatment. Up to 160 different bacteria species were found – some of them for the first time in vineyard soils. Total bacterial counts under herbicides were on average 260% higher than under mechanical weeding; however due to high variability this was not statistically significant. We suggest that herbicide-induced alterations of soil microorganisms could have knock-on effects on other parts of the grapevine system.

With an increasing intensification of viticulture, chemical weed control within and between grapevine rows is more widely used (Keller 2015). Weeds compete with vines for water and nutrients and herbicides are used to avoid trunk damage caused by mechanical weeding machinery and to reduce working time spent in the vineyard. Among the most often used herbicides in vineyards are those based on the active ingredients glyphosate, glufosinate and flazasulfuron (Bauer et al. 2017). While effects on soil organisms of fungicides and/or insecticides have been reported from vineyards (Paoletti et al. 1998), very little is known on the impacts of herbicides (Zaller et al. 2018). Moreover, laboratory and pot studies showed various non-target effects of herbicides on soil microbial communities (van Hoesel et al. 2017; Wilkinson and Lucas 1969; Zaller et al. 2014).

Weed control measures can affect soil microorganisms either by eliminating weeds and their associated rhizosphere or by directly influencing the physiology and diversity of microorganisms (Carson et al. 2007; Corneo et al. 2013; Marilley and Aragno 1999). Studies are mainly concerned with bacterial communities; the few that studied fungi focused mainly on arbuscular mycorrhizal symbiosis in arable fields (Rillig 2004). Only a few studies investigated soil microbial communities in vineyards (Fernández-Calviño et al. 2010; Likar et al. 2017; Samad et al. 2017; Steenwerth et al. 2008; Zaller et al. 2018). A study reports significant different microbiomes between grapevines roots and rhizosphere and weeds (Samad et al. 2017).

For healthy and fertile vineyard soils, a diverse population of microorganisms is essential for plant health, plant growth and productivity (Chaparro et al. 2012; Zarraonaindia et al. 2015). Knowledge of interactions between microorganisms in the soil, the rhizosphere and the phyllosphere is increasing (Pinto and Gomes 2016). Bacterial communities on the grapes have been shown to influence the organoleptic properties of the wine, what contributes to a regional terroir (Mezzasalma et al. 2017). Additionally, management practices as well as ecological and environmental factors influence soil microbiota (Pancher et al. 2012; Zehetner et al. 2015), thereby affecting the characteristics of the wine (Zarraonaindia et al. 2015).

The objective of the current study was to examine potential effects on soil microorganisms of three commonly used herbicides in comparison to mechanical weeding. The three herbicides differed in their mode-of-action. Herbicide one contained the active ingredient glyphosate that acts on the 5-enolpyruvylshikimate-3-phosphate (EPSP) synthase in plants (Steinrücken and Amrhein 1980). Herbicide two contained glufosinate that acts on glutamine synthetase (Duke 2014). Herbicide three was based on flazasulfuron that inhibits the amino acid synthesis, cell division and ultimately plant growth (Magné et al. 2006). Studies on herbicide effects on soil microbial communities can help developing more sustainable weed control measures for vineyard management.

## Materials and Methods

The study was conducted in 2016 in an experimental vineyard (Rothäcker XV) of the Federal College and Research Center for Viticulture and Pomology, in Klosterneuburg, near Vienna, Austria (coordinates 48.294809ºN, 16.324693ºE; 192 m above sea level). The vineyard consisted of 22, on average 30 m long vineyard rows and was established in 2011 with the white grape variety Gewürztraminer (*Vitis vinifera* L.) using trellis (grapevine within-row distance: 1.0 m; row distance: 2.8 m). The area is south-facing, slightly inclined and the inter-rows were cultivated according to the Austrian soil erosion prevention programme allowing only tillage of every second inter-row, while leaving the other rows uncultivated and vegetated (ÖPUL 2007). The vineyard was organically fertilised once (23 March 2016, 61 kg N/ha; product BioAgenasol, Agrana, Austria); plant protection measures were applied evenly across the vineyard following good viticultural practice (Table 1). Soils at the study site developed from alluvial soils of sandy, brown primary material and rounded pebble stones; additionally, chiselled Flysch marl stemmed from colluvial processes.

**Table 1:** Plant protection applied additionally to the weed control treatments in the study vineyard during the course of the experiment. Soil samples were taken on 23 June 2016

Date 2016	Pest/disease	Product	Dosage	Manufacturer
13 April	Grape leaf rust mites	Thiovit Netzschwefel	3.0 kg/ha	Syngenta Agro
22 April	Powdery mildew	Thiovit Netzschwefel	2.0 kg/ha	Syngenta Agro
		Vivando	0.15 L/ha	BASF SE
	Downy mildew	Polyram WG	0.8 kg/ha	BASF SE
	Grape black rot Dead arm			
9 May	Powdery mildew	Thiovit Netzschwefel	2.0 kg/ha	Syngenta Agro
		Vegas	0.25 L/ha	Nisso Chemical Eur
	Downy mildew	Polyram WG	0.8 kg/ha	BASF SE
	Grape black rot			
	Dead arm			
1 June	Powdery mildew	Thiovit Netzschwefel	2.0 kg/ha	Syngenta Agro
		Flint max	0.18 kg/ha	Bayer Crop Science
	Downy mildew	Dithane Neo Tec	2.0 kg/ha	Star Agro
	Rotbrenner disease			
	Dead arm			
18 June	Powdery mildew	Thiovit Netzschwefel	2.0 kg/ha	Syngenta Agro
		Vegas	0.5 L/ha	Nisso Chemical Eur
	Downy mildew	Aktuan gold	1.56 kg/ha	BASF SE
		Cuprofor flow	1.0 L/ha	Kwizda Agro

We selected two rows for each herbicide treatment. Each herbicide treatment covered the undergrowth of five grapevines at a width of 0.5 m; a distance of two untreated grapevines was left between herbicide applications. Between herbicide treated rows two rows were left untreated to avoid cross-contamination.

We used four different treatments for weed control within grapevine rows: three broadband herbicides with different active ingredients and mechanical weeding as a control. All herbicides were applied according to good farming practice at the recommended dosage. Roundup PowerFlex (Monsanto Agrar Deutschland, Düsseldorf, Germany) with the active ingredient glyphosate as potassium salt (200 g/L) was applied in a concentration of 3.75 L/ha. Basta 150 SL (Bayer CropScience Austria, Vienna, Austria) based on glufosinate-ammonium was applied at 5.0 L/ha. Katana (ISK Biosciences Europe, Brussels, Belgium) based on flazasulfuron was applied at 200 g/ha.

Herbicides were applied by an experienced viticultural technician early in the morning with a backpack sprayer on 7 April 2016 first at a temperature of 12°C under calm conditions. Mechanical weeding was also performed on 7 April 2016 using a hand weeding tool; no herbicides were applied between rows. As recommended by the manufacturer, Basta was applied a second time (7 June 2016). Vegetation height at the time of weed control applications was about 20 cm.

Two bulk soil samples per treatment replicate were taken on 23 June 2016 (77 days after the first herbicide application) from the middle of the treated rows in 20 cm distance to the grapevine at 10–20 cm depth using a quadratic soil corer (5 × 5 × 10 m, length × width × depth).

For determination of microbial counts and identifying the microorganisms 1 g of each soil sample was randomly selected and used for a dilution series. As nutrient solution three different agar plates were used, Malt Extract Agar MEA (Weidenbörner 1998), Wallerstein Laboratory WL and Tryptic Soy Agar TSA (Carl Roth GmbH, Karlsruhe, Germany). After 6 days of incubation at 24°C the microbial colonies were assessed by visually differentiating bacteria, yeasts and molds.

Yeasts were purely cultivated on MEA agar and cultivated in Malt Extract bouillon for 4–7 days. Then yeasts were reamed with a mortar and purified with a MasterPureTM-Purification Kit (MCD85201 Epicentre, Illumina Company, USA). Afterwards an ITS1–ITS4 PCR (White et al. 1990) was performed. Bacteria were purely cultivated on PC agar and cultivated overnight in Standard I nutrient bouillon (art. 1.07882.0500, Merck, Darmstadt, Germany). DNA purification was carried out with MasterPureTM - purification kit (MCD85201 Epicentre, Illumina Company, USA) followed by a PCR with AC1 and AC3 primer (Poblet et al. 2000).

For gel band purification a WizardSV Gel and PCR Clean–Up System (A 9281, Promega, USA) was used. Sequencing was performed by an external laboratory (Eurofins Genomics GmbH, Ebersberg, Germany). DNA results were analysed using the database of the US National Center for Biotechnology Information (https://www.ncbi.nlm.nih.gov/).

From the same soil samples, about 400 g of fresh soil was sent to a commercial laboratory (Eurofins Genomics, Ebersberg, Germany) in order to perform next-generation sequencing (NGS). The DNA-extraction was performed using a NucleoSpin soil kit (Macherey Nagel, Düren, Germany). Analyses were made with an Illumina MiSeq v3, 2 × 300 bp Modus. For the target region V1V3 the primers V1V3_F: AGAGTTTGATCATGGCTCAG and V1V3_R: GTATTACCGCGGCTGCTG were used. The applied PCR program involved 2 min 95°C, then 28 cycles (30 s 95°C + 50 s 50°C + 1 min 72°C), then 6 min 72°C and finally 4°C. The genetical sequences were attached to the associated 16S region. The taxonomic comparison was done with the software QIIME (version 1.8.0, http://qiime.org) and the NCBI database. After preprocessing and quality filtering 2,408,208 16S-gen sequences with a range from 127,194 to 566,193 sequences per sample were gained. Diversity of microbial communities from NGS data were analysed by calculating Shannon diversity and evenness indices on the taxonomic categories phyllum, class, order, family, genus and species.

Prior to the microbiome analysis, raw reads were demultiplexed/debarcoded based on the unique forward and reverse sequencing indices and/or inline-barcode sequences. To preserve only high-quality reads, all reads with sequencing errors or reads with ambiguous bases (“N”) were removed. Indices/barcodes as well as primer sequences were clipped from the reads. The remaining set of high-quality reads was processed using minimum entropy decomposition (Eren et al. 2015). Minimum entropy decomposition (MED) provides a computationally efficient means to partition marker gene datasets into OTUs (Operational Taxonomic Units). Each OTU represents a distinct cluster with significant sequence divergence to any other cluster. By employing Shannon entropy, MED uses only the information-rich nucleotide positions across reads and iteratively partitions large datasets while omitting stochastic variation. The MED procedure outperforms classical, identity-based clustering algorithms. Sequences can be partitioned based on relevant single nucleotide differences without being susceptible to random sequencing errors. This allows a decomposition of sequence data sets with a single nucleotide resolution. Furthermore, the MED procedure identifies and filters random “noise” in the dataset. This includes singletons and putative chimeric sequences. To assign taxonomic information to each OTU, BLAST alignments of cluster representative sequences to the NCBI sequence database were performed. A most specific taxonomic assignment for each OTU was then transferred from the set of best-matching reference sequences. Hereby, a sequence identity of 80% across at least 80% of the representative sequence was a minimal requirement for considering reference sequences. Further processing of OTUs and taxonomic assignments was performed using the QIIME software package (version 1.8.0, http://qiime.org/). Abundances of bacteria and archaea taxonomic units were normalized using lineage-specific copy numbers of the relevant marker genes to improve estimates (Angly et al. 2014). Results of read preprocessing, OTU picking, and taxonomic assignment is presented in Table 2.

**Table 2:** Summary of next generation sequencing preprocessing

Parameter	OTUs	Percentage
Total number of input sequences	2,411,541	100.0
Remaining sequences after preprocessing and quality filtering	2,408,208	99.9
Total number of sequences assigned to OTUs	1,851,837	76.8
Total number of sequences assigned to taxa	1,284,409	53.3
Copy-number corrected total count	685,031	N/A
Total number of OTUs	6957	100.0
Number of OTUs assigned to taxa	4728	68.0
Sequences per sample assigned to OTUs		
Min	99,429	
Max	432,715	
Median	144,058	
Mean	231,479	
Std. dev.	145,993	

The number of OTUs correlates with the diversity of the data set. Sequences that were considered as noise by the OTU picking algorithm were not assigned to an OTU. This includes singletons and putative chimeric sequences. The fraction of OTUs that could be assigned to taxa indicates how well the microbiome is represented in the used reference database. A copy-number correction was performed for bacterial species only (Angly et al. 2014). To do so, the number of reads assigned to a species was divided by the known or assumed copy-number of marker genes/regions. After preprocessing, sequences were clipped to 255 bp length to remove low quality bases from the 3′ end and to ensure that all sequences have the same length. The latter is crucial for the MED analysis.

Statistical analysis of CFUs and OTUs were performed using the FASTQ file format (Cock et al. 2010) within the software package R (version 3.0.2, The R Foundation for Statistical Computing 2013). Shannon- and Evenness indices were analysed using one-way analysis of variance (ANOVA) with the factor herbicide treatment (4 levels). Mean comparisons between herbicide treatments were performed using Tukey tests. Differences with *p* < 0.05 were considered to be significant.

## Results and Discussion

**Fig. 1:**
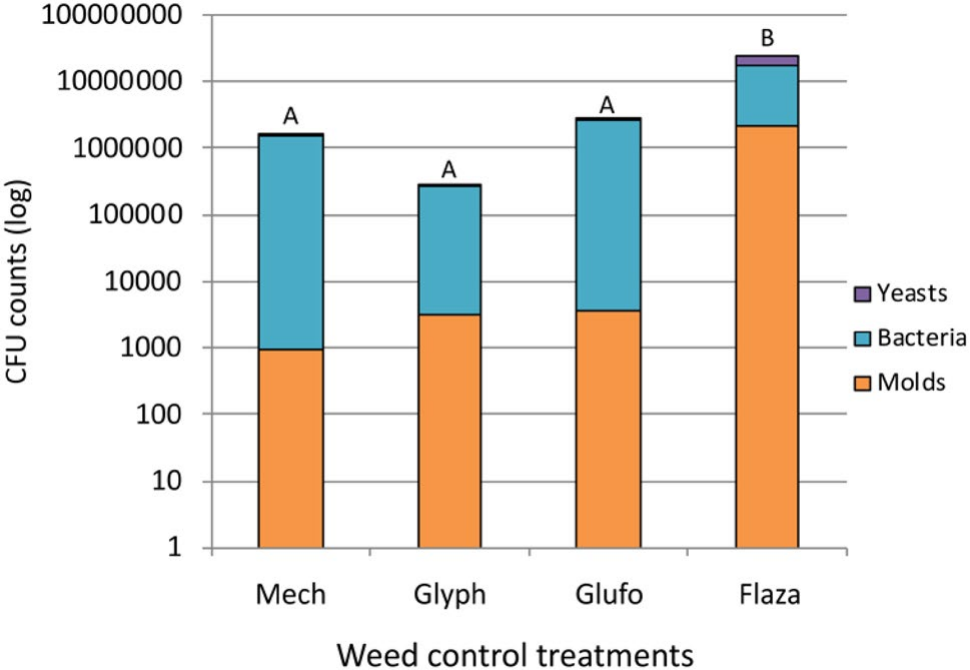
Mean proportions of yeasts, bacteria and molds in soil samples in vineyards rows after mechanical weeding (mech) or three herbicide treatments (glyph…glyphosate, glufo…glufosinate, flaza…flazasulfuron). Different letters denote significant differences of the total CFU counts (Tukey HSD, *p* < 0.05). Data obtained by classical sequencing

Weed control significantly affected total CFUs in vineyard soils. Significantly more total CFUs were found under flazasulfuron (24.13 ± 83.32 × 10^6^, mean ± SD) than under glyphosate (0.27 ± 0.79 × 10^6^), glufosinate (2.78 ± 9.32 × 10^6^) or the control treatment (1.56 ± 5.48 × 10^6^; Fig. 1). Weed control treatments had no significant effect on the proportion of yeasts, molds and bacteria in soil samples (Fig. 1). There was a trend for considerably more molds and yeasts but less bacteria under flazasulfuron than under the other treatments, however due to high heterogeneity this was not statistically significant. Such unclear patterns of herbicide effects on the microbial soil communities have also been reported by others (Kopčáková et al. 2016; Newman et al. 2016). However, a clear difference in soil bacterial and fungal composition in vineyards was reported between herbicide treated and untreated grapevine rows (Chou et al. 2018; Hendgen et al. 2018).

**Fig. 2:**
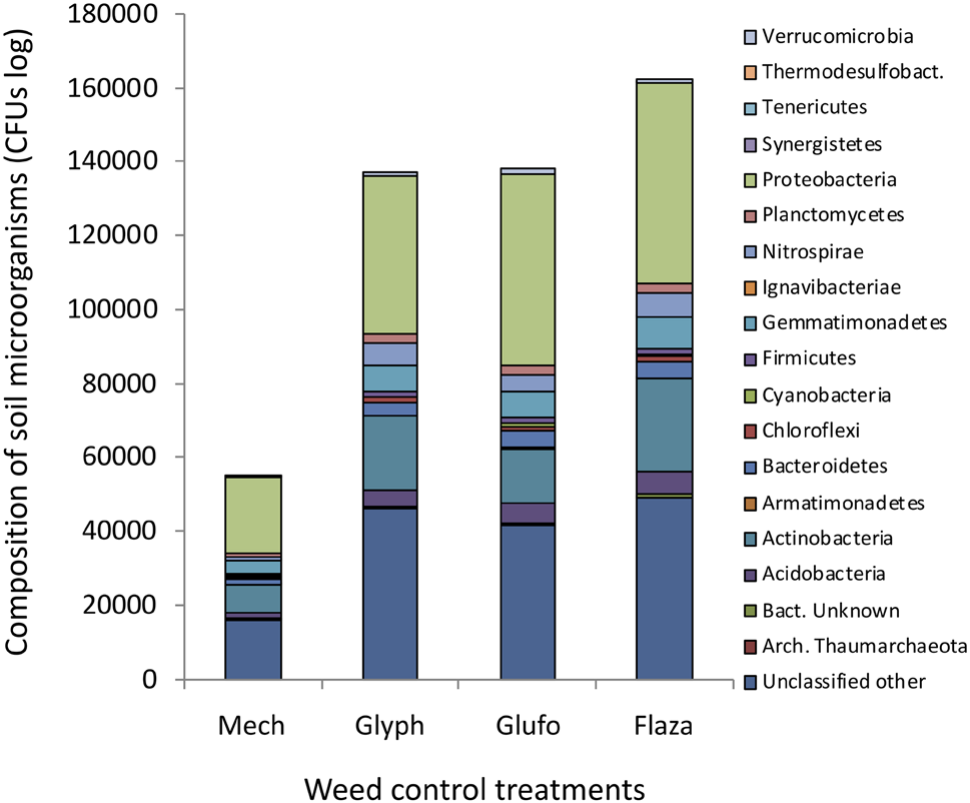
Composition of soil bacteria and archeobacteria communities in vineyards rows after mechanical weeding (mech) and three herbicide treatments (glyph…glyphosate, glufo…glufosinate, flaza…flazasulfuron). Data obtained by NGS sequencing

NGS analyses showed that abundances of cultivable and not-cultivable soil bacteria under herbicide treatments were on average 264% higher than under mechanical weeding (Fig. 2). However, due to high data variability this was not statistically significant. Across treatments we found strains of the following taxa with decreasing abundances: Proteobacteria (35.8% ± 3.6%, mean ± SD across treatments), Actinobacteria (13.0% ± 2.4%), Gemmatimonadetes (5.5% ± 1.0%), Acidobacteria (3.5% ± 0.5%), Nitrospira (3.4% ± 0.9%), Bacterioidetes (3.1% ± 0.6%), Ignavibacteriae (2% ± 0.1%), Plantomycetes (1.7% ± 0.3%), Firmicutes (1.2% ± 0.2%), Chloroflexi (1.0% ± 0.2%), Verrucomicrobia (0.9% ± 0.3%), Cyanobacteria (0.2% ± 0.1%), Armatimonadetes (0.1% ± 0.1%), Synergistes (0.005% ± 0.003%), Tenericutes (0.003% ± 0.003%), Thermodesulfobacteria (0.02% ± 0.02%) and unclassified strains (30.6% ± 2.3%).

**Fig. 3:**
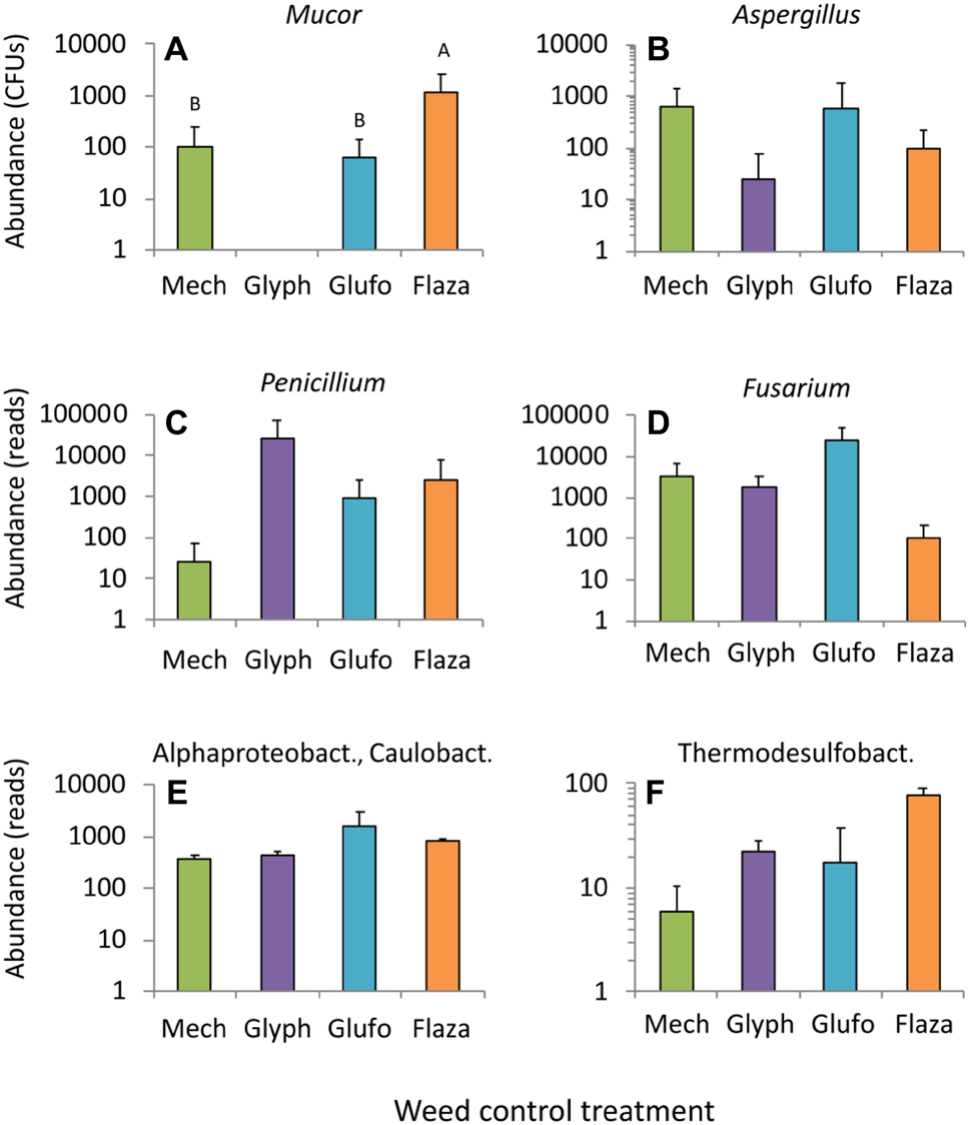
Selected taxa of soil microorganisms in vineyards rows under mechanical weeding (mech) and application of three herbicide (glyph…glyphosate, glufo…glufosinate, flaza…flazasulfuron). Means ± SD. Different letters denote significant differences (Tukey HSD, *p* < 0.05). Fungi data obtained using classical sequencing, bacteria data by NGS sequencing

Considering individual fungal taxa, only the abundance of *Mucor* was significantly affected by herbicide treatments (Fig. 3a). *Mucor* was absent under glyphosate and highest under flazasulfuron with similar CFUs under mechanical weeding and glufosinate (Fig. 3a). *Mucor* spp. are very common mainly in organic plant material like compost, fruits or vegetable, wood pellets and farmyard manure and are known to cause food spoilage (Domsch et al. 2007; Hoog et al. 2000). *Mucor* has been detected in air samples in Italian vineyards (Magyar et al. 2009), in grape berries in the Tokaj wine region in Hungary (Felšöciová et al. 2015) and is described as a pathogenic fungus on pear fruits (Mari et al. 2000). To the best of our knowledge the current study is among the first describing *Mucor* from vineyard soil samples below 10 cm depth.

The ubiquitous *Aspergillus* sp., *Penicillium* sp. and *Fusarium* sp. were detected in all treatments with high variability (Fig. 3b–d; Table 3), however no significant effect of weed control was seen. Alphaproteobacteriaceae and Caulobacteriaceae were observed in all samples but with little variation among treatments (Fig. 3c). Presence of Caulobacterales (Fig. 3e) have been described in the Chardonnay and Merlot varieties (Campisano et al. 2014; Pinto and Gomes 2016) and in grapevine roots (Samad et al. 2017) and in association with copper (Andreazza et al. 2012). In the current study there was a not-significant trends of 1%–37% higher reads of *Caulobacter* under herbicides than under mechanical weeding. Also, Thermosulfobacteria showed a not-significant trend to 9%–58% higher reads under herbicide treatments than under mechanical weeding (Fig. 3f). Thermodesulfobacteria are sulfate-reducing usually found in hot springs (Meyer-Dombard and Amend 2014; Wang et al. 2013) and were described after treatment with *Autographa californica* multiple nucleopolyhedovirus (Fu et al. 2015) or after biochar application (Abujabhah et al. 2017).

**Table 3:** Presence (+) or absence (−) of particular fungi taxa in soil samples under mechanical weeding (mech) and application of three herbicides (glyph…glyphosate, glufo…glufosinate, flaza…flazasulfuron)

Fungi taxa	Mech	Glyph	Glufo	Flaza
*Acremonium* sp.	–	–	+	–
*Arthroderma* sp.	–	–	–	+
*Aspergillus* sp.	+	+	+	+
*Cladosporium* sp.	–	+	+	–
*Clonostachys rosea*	+	–	–	–
*Colletotrichum* sp.	–	+	–	–
*Cunninghamella* sp.	–	+	–	–
*Dipodascus* sp.	+	–	–	–
*Fusarium* sp.	+	+	+	+
*Gongronella butleri*	–	–	+	–
*Mortierella* sp.	–	+	–	–
*Mucor* sp.	+	–	+	+
*Paecilomyces marquandi*	–	–	+	–
*Penicillium* sp.	+	+	+	+
*Scedosporium* sp.	–	+	–	–
*Sporothrix* sp.	–	–	+	–
*Striatibotrys* sp.	–	+	–	+
*Trichoderma* sp.	+	+	–	–

Taxa in alphabetical order

Changes in bacterial reads were reported in the rhizosphere of glyphosate-tolerant of corn (*Zea mays*) and soybean (*Glycine max*) in response to glyphosate treatment (Newman et al. 2016).

Many fungi described in the current study are reported from vineyard soils for the first time, mainly because of the use of modern sequencing methods (Morgan et al. 2017; Wei et al. 2018). Several taxa were also found specifically under certain treatments. Thus, for the sake of clarity we present an overview of presence/absence data of soil microorganisms found in different weed control treatments (Table 3). However, given the fact that our study only covers one field season in one experimental vineyard we decided for a cautious interpretation of the findings. Overall, it has to be noted that there is a great lack of knowledge on specific functions for most taxa in soil. More long-term studies are needed to assure the generality of our findings.

Under mechanical weeding only the yeast species *Clonostachys rosea* and the saprophytic fungi *Diapodascus* sp. were found while they were absent under herbicide treatments (Table 3). *Clonostachys rosea* is widespread occurring in the soil and in rotten plants and can suppress spores of botrytis bunch rot (Dong et al. 2004; Morandi et al. 2003) and can be pathogens against yeasts (Li et al. 2006; Zhao et al. 2005). Chemical isolates of *C. rosea* also showed a negative effect on the nematodes *Caenorhabditis elegans, Panagrellus redivivus*, and *Bursaphelenchus xylophilus* (Zhang et al. 2008). Species of the family Dipodascaceae are saprophytically in the plant sap of trees (e.g., *Dipodascus albidus*) or are colonizing dead insects (Cannon and Kirk 2007).

Under glyphosate only the fungi *Colletotrichum* sp., *Cunninghamella* sp., *Mortierella* sp. and *Scedosporium* sp. were found (Table 3), suggesting that herbicide-specific nutrients favors these fungi. Many *Colletotrichum* species are plant pathogenic and have a mutualistic relationship with their host plant (Rodriguez and Redman 2008). For example *Colletotrichum coccodes* causes tomato anthracnose on the fruit, black dots on the roots and blemishes on the surface of potatoes (Hughes 1958). *Cunninghamella* sp. is affected by fertilization and can be suppressed by *Stachybotrys* sp. (= *Striatibotrys* sp.) and *Trichoderma viride* (Domsch et al. 2007). *Mortiella* sp. is together with *Mucor* one of the first yeast taxa growing on roots of debilitated plants (Deacon 2005; Salt 1977; Webster 2012).

Under glufosinate only the fungi *Acremonium* sp., *Gongronella butleri, Paecilomyces marquandi* and *Sporothrix* sp. were found (Table 3). The genus *Acremonium* covers more than 100 species living saprophytically on dead plant material or in the soil. Many species can cause diseases in humans (Domsch et al. 2007; Fincher et al. 1991; Hoog et al. 2000). *Gongronella butleri* is one of the most important species of the Zygomycetes class also used for industrial production (Bartnicki-Garcia 1968; Tan et al. 1996). *Paecilomyces marquandi* can be detrimental to nematodes and can cause allergies in humans (Kilama et al. 2007; Mücke and Lemmen 2005). Additionally, it contains leucinostatine having an antimicrobial effect against grampositive bacteria and many fungi (Fukushima et al. 1983a, b). The fungi *Sporothrix* sp. can be found in the soil and on decomposing plant material and can induce infections in humans and animals (Barros et al. 2011; de Meyer et al. 2008; Vasquez-del-Mercado et al. 2012).

Under flazasulfuron only the yeast *Arthroderma* sp. were exclusively found (Table 3). Not much is known about the role of *Arthroderma* in ecological systems, however it may cause skin problems in humans (Hiernickel et al. 2016).

Several taxa were found under more than one treatment (Table 3). Species of the genus *Cladosporium* are very common and mainly occur in the soil and on plants, we found *Cladosporium* sp. under glyphosate and glufosinate. The fungi genus *Trichoderma* was found under mechanical weeding and under glyphosate and is normally occurring in all soil types is representing the most commonly cultivated fungi also forming avirulent symbioses with plants (Harman et al. 2004). The mold *Striatibotrys* sp. (also called *Stachybotrys* sp.) produces the stachybotrys mycosis pathogen (Summerbell et al. 1989) and was found under glyphosate and flazasulfuron.

This is among the first field studies investigating the effects of three commonly used herbicides and mechanical weeding on soil microorganisms in vineyards. As all treatments left weed material on ground, the finding that certain herbicides stimulated or suppressed certain fungi suggests that herbicide-specific active ingredients, adjuvants or nutrients might be responsible for this effect. Although the study vineyard was evenly treated with other pesticides according to good viticultural practice, potential interactions with specific herbicides are likely. It is important to note that the findings need to be interpreted with caution as the study was conducted during one field season in one experimental vineyard. Overall, we know very little about the role of microorganisms in vineyard soils and their consequences for health, yield and quality of the grapevine (Belda et al. 2017). However, the microbial terroir concept suggests important association between microorganisms in different compartments of the vineyard ecosystem and wine characteristics (Bokulich et al. 2014; Gilbert et al. 2014; Miura et al. 2017). Clearly, more long-term studies are needed to further elucidate non-target effects of pesticides used in vineyard management.
